# Supplementary material for: Automatic landmark correspondence detection in medical images with an application to deformable image registration
Source: J Med Imaging (Bellingham). 2023 Feb 25;10(1):014007. doi: 10.1117/1.JMI.10.1.014007 (PMC9957682; doi:10.1117/1.JMI.10.1.014007)
Supplement: Supplementary file 1 [file JMI_010_014007_SD001.pdf]

Supplementary Material

Monika Grewal, Jan Wiersma, Henrike Westerveld,
Peter A. N. Bosman, Tanja Alderliesten

Journal: Journal of Medical Imaging
Manuscript title: Automatic Landmarks Correspondence Detection in Medical Images with an Application to Deformable Image Registration

Retrospective Analysis

The extent of the added value provided by the use of automatic landmark correspondences in DIR was lower in the clinical deformations test set as compared to the simulated deformations test set. Therefore, we analyzed the TRE values of each manual landmarks in the clinical deformations test set to understand the possible causes for the lack of performance gain by using automatic landmarks in DIR. Specifically, we calculated the number of automatic landmarks

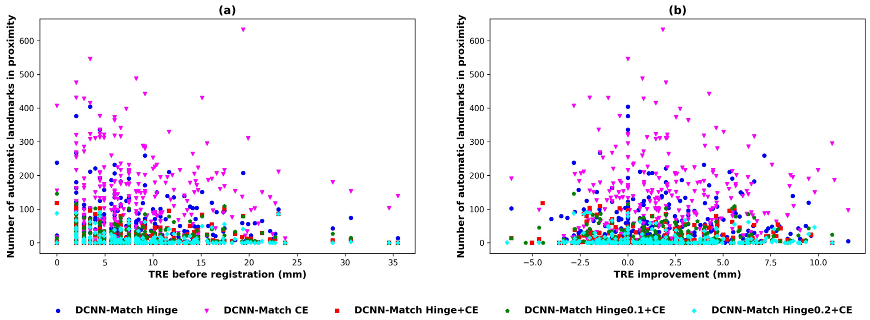

Figure S1: **Results on clinical deformations test set.** For each manual landmark, the number of automatic landmark correspondences predicted in 16 mm proximity to that manual landmark has been plotted against (a) the corresponding TRE_{before} value and (b) TRE improvement value as obtained by subtracting TRE_{after} value from TRE_{before} value.

in proximity (16 mm) to each of the manual landmark. We plotted this value against the TRE_{before} value (representative of the underlying deformation in that region) of that manual landmark (shown in Figure S1 (a)). The plot shows that automatic landmarks were predicted in the regions of high deformation as

well, especially by DCNN-Match CE. Therefore, a lack of the presence of automatic landmarks in highly deformed regions could not be the sole cause for the lack of performance gain in DIR.

Further, we calculated the TRE improvement for each manual landmark by subtracting TRE_{after} from TRE_{before} values. A positive high number indicates higher improvement in TRE values (or DIR performance). In Figure S1 (b), the TRE improvement values have been plotted against the number of automatic landmarks in proximity for each manual landmark. We observed that the TRE value of some of the manual landmarks in some of the patients did not improve despite the presence of automatic landmarks in their proximity.

In conclusion, the above analysis shows that a straightforward pattern regarding the spatial distribution of automatic landmarks relative to the manual landmarks cannot be established in case of clinical deformations. Consequently, a direct relationship between the quality of automatic landmark correspondences and the DIR performance cannot be established.
